# Lentinan administration alleviates diarrhea of rotavirus-infected weaned pigs via regulating intestinal immunity

**DOI:** 10.1186/s40104-021-00562-6

**Published:** 2021-03-09

**Authors:** Xiangqi Fan, Haiyan Hu, Daiwen Chen, Bing Yu, Jun He, Jie Yu, Junqiu Luo, Erik Eckhardt, Yuheng Luo, Jianping Wang, Hui Yan, Xiangbing Mao

**Affiliations:** 1 Key Laboratory for Animal Disease-Resistance Nutrition of China Ministry of Education, Key laboratory of Animal Disease-resistant Nutrition and Feed of China Ministry of Agriculture and Rural Affairs, Key laboratory of Animal Disease-resistant Nutrition of Sichuan Province, Institute of Animal Nutrition, Sichuan Agricultural University, No. 211, Gongpinghuimin Road, Wenjiang District, Chengdu, 611130 Sichuan Province People’s Republic of China; 2 Adisseo SAS, Center of Excellence and Research in Nutrition, 03600 Malicorne, France

**Keywords:** Gut immunity, IPEC-J2 cells, Lentinan, Rotavirus, Weaned pigs

## Abstract

**Background:**

Lentinan (LNT) may regulate many important physiological functions of human and animals. This study aimed to verify whether LNT administration could relieve diarrhea via improving gut immunity in rotavirus (RV)-challenged weaned pigs.

**Methods:**

Twenty-eight weaned pigs were randomly fed 2 diets containing 0 or 84 mg/kg LNT product for 19 d (*n* = 14). RV infection was executed on d 15. After extracting polysaccharides from LNT product, its major monosaccharides were analyzed. Then, LNT polysaccharide was used to administrate RV-infected IPEC-J2 cells.

**Results:**

Dietary LNT supplementation supported normal function of piglets even when infected with RV, as reflected by reduced growth performance loss and diarrhea prevalence, and maintained gut immunity (*P* < 0.05). The polysaccharide was isolated from LNT product, which molecular weight was 5303 Da, and major monosaccharides included glucose, arabinose and galactose. In RV-infected IPEC-J2 cells, this polysaccharide significantly increased cell viability (*P* < 0.05), and significantly increased anti-virus immunity via regulating pattern recognition receptors and host defense peptides (*P* < 0.05).

**Conclusion:**

Those results suggest that LNT administration increases the piglets’ resistance to RV-induced stress, likely by supporting intestinal immunity.

**Supplementary Information:**

The online version contains supplementary material available at 10.1186/s40104-021-00562-6.

## Background

Shiitake mushroom can improve the immunologic function, and has been used to prevent and treat various diseases of humans and animals for thousands of years in China and Japan [1]. Lentinan (LNT) is one of the major compounds purified from this mushroom [2]. Many researchers have reported that lentinan has antitumor, anti-inflammatory, antioxidant and antiviral functions via improving immunity [3–6].

Rotavirus (RV) is a major pathogen that induces severe diarrhea in infants and young animals. After ingestion, RV particles invade the non-dividing mature enterocytes of small intestine, induce the inflammatory response, and even cause dysfunction of immune system in the children and weaned pigs [7–9]. Recently, RV vaccine has also been studied by many researchers, and there are some effective vaccines in some species. However, in some developing countries, these vaccines are lack of effectiveness because of malnutrition, and specific storage conditions [10, 11]. Our previous study has shown that LNT administration can reduce RV-infected diarrhea, and improve intestinal barrier function [12]. Then, based on the capacity of enhancing immunity, it is possible that LNT administration improving RV-induced diarrhea also be associated with immune function.

Therefore, via the *in vivo* and *in vitro* experiments, this study was conducted to test the hypothesis that LNT administration might improve growth performance and resilience in RV-infected piglets. Therefore, in this process, the effect of intestinal immune function will also be analyzed.

## Methods

### Animal trial

#### Animals and diets

The experimental protocol was approved by Animal Care Advisory Committee of Sichuan Agricultural University. A total of 28 twenty-eight-day-old weaning pigs (Duroc × Landrace × Yorkshire) with an initial average BW of 7.51 ± 1.37 kg were individually housed in metabolic cages (1.5 m × 0.7 m × 1.0 m). The pigs were fed with the experimental diets 4 times daily at 08:00, 12:00, 16:00 and 20:00, and could have access to water *ad libitum*.

The basal diet was formulated to meet NRC (2012) [13] nutrient recommendations for pigs (7–11 kg). Its composition and nutrient levels were shown in Supplementary Table 1. The LNT diet was formed by supplementing 84 mg/kg LNT product in the basal diet. This LNT product was purchased from Sichuan Hengruitongda Biotechnology Co. Ltd. The active components (lentinan) accounted for 30% of this LNT product.

#### Experimental design and sample collection

Following 3 d of acclimatization, all pigs were divided randomly into two groups on basis of initial body weight and litter origin (*n* = 14), and were fed with the basal and LNT-supplemented diets, respectively. The experimental duration was 19 d. From the 12th to 14th day, nutrient digestion was estimated as described previously [14].

RV preparation and virus titre determination [tissue culture infective dose 50 (TCID_50_) value] were executed as described previously [15].

On the 15th day, all pigs were infused 5 mL of sterile 100 mmol/L NaHCO_3_ solution. Then, half of pigs in two groups were gavaged orally with 4 mL (10^6^ TCID_50_/mL) of RV, whereas the other pigs received 4 mL of the sterile essential medium by oral gavage. Following RV infusion, the diarrhea symptom of all pigs was observed and recorded in each day. Fecal consistency was scored: 0, normal; 1, pasty; 2, semiliquid; 3, liquid. These were used to calculate diarrhea index, which would represent diarrhea severity [16–18]. Diarrhea happened when fecal consistency score of pigs is ≥2 [19]. Average daily gain (ADG), average daily feed intake (ADFI) and feed conversion of all pigs were calculated via measuring feed intake in each day and body weight on d 1, 15 and 20.

On the 20th day, after 12 h starvation, all pigs were weighted. Blood samples of all pigs were collected from jugular vein, centrifuged at 3500 × *g* for 10 min, and serum was gathered. Then, the pigs were refed for 1.5 h. Following refeeding, all pigs were euthanized by intracardially injecting with Na pentobarbital (50 mg/kg of body weight) and exsanguinated. The jejunum was immediately separated, and cleaned with ice-cold saline. Jejunal mucosa were gathered by scraping the gut wall with a glass microscope slide, frozen in liquid nitrogen, and stored at − 80 °C until analysis.

#### Analysis of nutrient digestibility

Ash insoluble in hydrochloric acid was used as non-absorbable digestion marker in the digestion trial. Dry matter (DM), crude protein (CP), ether extract (EE), calcium (Ca), phosphorus (P), gross energy (GE) and ash insoluble in hydrochloric acid in feces and feeds were analyzed on the basis of the method of Diao et al. [14]. Nutrients digestibility was calculated as [100–100 × (A × D) / (B × C)]; A and B are the content of ash insoluble in hydrochloric acid in feeds and feces, respectively; C and D are the content of nutrients in feeds and feces, respectively.

#### Analysis of urea nitrogen (UN), rotavirus antibody (RV-ab), rotavirus non-structural protein 4 (NSP4), immunoglobulin (Ig) and/or interferon-beta (IFN-β) levels in serum and jejunal mucosa

Jejunal mucosa (about 100 mg) were added to ice-cold PBS, shattered at 4 °C, and then centrifuged at 5000 *× g* for 15 min at 4 °C. The supernatant was used to measure the levels of RV-Ab (IgM), NSP4, secretory immunoglobulin A (sIgA) and IFN-β.

The serum UN level was measured with the kit (Nanjing Jiancheng Bioengineering Institute, Nanjing, China) according to the manufacturer’s instruction. The RV-Ab (IgM) and NSP4 concentrations in serum and/or jejunal mucosa were detected by using ELISA kit (TSZ ELISA, Framingham, MA, USA). Serum IgA, IgG and IgM concentrations, and the concentrations of sIgA and IFN-β in the jejunal mucosa were detected with ELISA kits from Nuoyuan Co. Ltd. (Shanghai, China).

#### Analysis of expressions of host defense peptides and some immune-related genes in the jejunal mucosa

Total RNA in the jejunal and ileal mucosa was isolated with TRIZOL reagent (TaKaRa Biotechnology (Dalian) Co., Ltd., Dalian, China) according to the manufacturer’s instructions. By using DU 640 UV spectrophotometer detection (Beckman Coulter Inc., Fullerton, CA, USA), the OD_260_:OD_280_ ratio of all samples ranged from 1.8 and 2.0, which indicated good quality RNA. The integrity of RNA was further analyzed by 1% agarose gel electrophoresis. Using RT Reagents (TaKaRa Biotechnology (Dalian) Co., Ltd., Dalian, China), RNA of all samples was reversely transcribed into complementary DNA according to the manufacturer’s instructions. The gene expression levels of porcine beta-defensin 1 (*pBD1*), *pBD2*, *pBD3*, Toll-like receptor 3 (*TLR3*), retinoic acid inducible protein 1 (*RIG-I*), melanoma differentiation-associated protein 5 (*MDA5*), mitochondrial antiviral signaling protein (*MAVS*), *IFN-β*, interferon stimulated gene 15 (*ISG-15*) and β-actin in jejunal mucosa were analyzed by real-time quantitative PCR using SYBR Premix Ex Taq reagents (TaKaRa Biotechnology (Dalian) Co., Ltd., Dalian, China) and CFX-96 Real-Time PCR Detection System (Bio-Rad Laboratories, Richmond, CA, USA) as described previously [20]. The primers of all genes, listed in Supplementary Table 2, were purchased by TaKaRa Biotechnology (Dalian) Co., Ltd. (Dalian, China). Relative gene expression to reference gene (β-actin) was determined in order to rectify the variance in amounts of RNA input in the reaction. Then, the relative gene expressions were calculated with the previous method [21].

#### Preparation of water-soluble polysaccharides

The water-soluble polysaccharides were prepared as previously described with some modifications [22]. Briefly, the LNT product (20 g) provided by Sichuan Hengruitongda Biotechnology Co. Ltd. was dissolved in 200 mL distilled water at 85 °C for 3 h. The supernatants were concentrated to 1/5 of the original volume in a rotary vacuum evaporator at 50 °C under reduced pressure. The proteins in supernatants were removed by adding the Sevag reagent (chloroform: n-butanol=4:1, v/v), and the liquid was dialyzed (3000 Da) with ultra-pure water for 48 h. Then, the solution was precipitated with 4 volume of 95% ethanol (v/v) at 4 °C for 24 h, and the precipitates were collected by centrifugation (2655×*g*, 10 min). Following lyophilization, the polysaccharides were produced. The purity of polysaccharides was measured according to previously published-methods [23].

#### Molecular weight analysis

The molecular weight (Mw) distributions were measured using high performance gel permeation chromatography (HPGPC) with an Agilent 1100 HPLC system equipped with a Waters 2410 refractive index detector and a TSK-GEL G5000 PWxL column (7.8 mm × 300 mm, TOSOH Co., Japan) as previously described [24]. The mobile phase consisted of ultrapure water which flowed at a rate of 0.8 mL/min and a temperature of 30 °C. The polysaccharide solution sample (20 μL, 2.0 mg/mL) was added in each run. The standard curve was created using Dextran standards (3.0 to 670 kDa, Sigma-Aldrich Co. LLC., USA).

#### Monosaccharide composition

The monosaccharide composition in polysaccharide samples was determined by using gas chromatography according to a previous method [24]. Briefly, the dried samples (10 mg) were hydrolyzed with 2 mL of trifluoroacetic acid (2 mol/L) at 110 °C for 2 h, and the solution was lyophilized after removing the excess acid. Then, hydroxylamine hydrochloride (10 mg) and pyridine (0.5 mL) were added to the hydrolysate, and this solution was incubated for 30 min at 90 °C. Following cooling to room temperature, acetic anhydride (0.5 mL) was added and reacted for 30 min in a water bath (90 °C). Standard monosaccharide samples [*D*-glucose, *D*-galactose, *L*-arabinose, *D*-mannose, *L*-rhamnose, and *D*-xylose (Sigma-Aldrich Co. LLC., USA)] were derivatized under the same conditions. The monosaccharide composition was identified by comparison with the retention times of monosaccharide standards.

### Cell culture trial

#### Cell culture

The IPEC-J2 cell line (porcine intestinal epithelial cells) was kindly supplied by Dr. Guolong Zhang at Oklahoma State University (Stillwater, OK, USA), and was cultured as described previously [19]. Briefly, IPEC-J2 cells were cultured with DMEM/F12 medium (Gibco Laboratories Life Technologies Inc., Grand Island, NY, USA) with 10% fetal bovine serum (Hyclone Laboratories Inc., Logan, UT, USA), 1% antibiotics (Penicillin-Streptomycin Solution, Hyclone Laboratories Inc., Logan, UT, USA) and 0.2% insulin-transferrin-selenium (Lonza, Walkersville, MD, USA) at 37 °C in 5% CO_2_.

#### Cell viability assay

The viability of IPEC-J2 cells was measured with the Cell Counting Kit-8 (CCK8; Beyotime, Jiangmen, China) according to the manufacturer’s instructions. In brief, IPEC-J2 cells were seeded in 96-well plates at 1.25 × 10^4^ cells/well. After 20 h, the varying concentrations (0.0, 1.5, 3.0, 6.0, 12.0 and 24.0 mg/L) of LNT polysaccharide were added to the cells (*n* = 9). At 6, 12 and 24 h following addition of LNT, CCK8 solution was added and incubated for 2 h. Cell viability was analyzed with a BioTek Synergy HT microplate reader (BioTek Instruments, Winooski, VT, USA) at an absorbance of 450 nm. This will get the suitable treating-dose and -time of LNT polysaccharide in following cell trials.

#### Cell experiments

The viability of IPEC-J2 cells that were supplemented with LNT polysaccharide and RV was also analyzed by CCK8. Briefly, IPEC-J2 cells were seeded in 96-well plates at 1.25 × 10^4^ cells/well. After 20 h, 0 or suitable dose of LNT polysaccharide were added to the cells for suitable exposure-time (*n* = 24). The media were removed, and cells were washed three times with PBS and incubated with media or RV [multiplicity of infection (MOI) = 3] for 1 h (*n* = 12). Following inoculum removal and washing twice with PBS, IPEC-J2 cells were supplemented with 0 or suitable dose LNT polysaccharide for 4 d. On d 1, 2, 3 and 4 d, CCK8 solution was added and incubated for 2 h. Cell viability was analyzed with a BioTek Synergy HT microplate reader (BioTek Instruments, Winooski, VT, USA) at an absorbance of 450 nm.

IPEC-J2 cells were seeded in 6-well plates at 2.5 × 10^5^ cells/well. Following 20 h, cells were exposed to the suitable dose and time of LNT polysaccharide at 37 °C in 5% CO_2_. Then, the media were removed, and cells were washed three times with PBS and incubated with RV (MOI = 3) for 1 h. After inoculum removal and washing twice with PBS, IPEC-J2 cells were supplemented with the suitable dose LNT polysaccharide for a further 3 d. All cells and supernatants were collected.

#### Analysis of NSP4 in cell supernatants

The concentration of NSP4 in cell supernatants was detected with a commercially available ELISA kit (TSZ, Framingham, MA, USA) according to the manufacturer’s instructions.

#### Analysis of gene expressions in IPEC-J2 cells

RNA was isolated from cells by TRIZOL reagent (TaKaRa Biotechnology (Dalian) Co., Ltd., Dalian, China) according to the manufacturer’s instructions. The methods of RNA concentration and integrity analysis, complementary DNA synthesis, real-time quantitative PCR and relative gene expression calculation were consistent with those in the animal experiment. The primers of all genes, listed in Supplementary Table 2, were purchased by TaKaRa Biotechnology (Dalian) Co., Ltd. (Dalian, China).

#### Statistical analysis

All experimental data were processed with Microsoft Excel 2013, analyzed by using SAS (version 8.1; SAS Institute, Gary, NC, USA), and indicated as means with their standard errors (SE). (i) Animal trial. The data of growth performance before RV challenge and nutrient digestibility in weaned pigs were analyzed with the unpaired *t* test. And except these and diarrhea rate, the other data were analyzed as a 2 × 2 factorial with the general linear model procedures of the Statistical Analysis Package. The model factors contained the effects of LNT (basal or LNT-supplemented diets), RV challenge (infusing essential medium or rotavirus), and their interaction. (ii) Cell-culture trial. The cell viability was analyzed with the unpaired *t* test. The other data were analyzed using one-way ANOVA, followed by Duncan’s Multiple Range test. The *P*-value less than 0.05 was deemed statistical significance while the *P-*value less than 0.10 was deemed statistical trends.

## Results

### Growth performance, SUN, and nutrient digestibility of weaned pigs

During the first 2 weeks, supplementing LNT in the diet reduced feed conversion of weaned pigs (*P <* 0.05, Table 1). RV challenge increased feed conversion and SUN (*P* < 0.05), and tended to reduce ADG (*P* = 0.08) in weaned pigs (Table 1). After RV infusion, LNT administration elevated ADG, and decreased feed conversion and SUN in weaned pigs (*P* < 0.05, Table 1). Moreover, in the RV-infected pigs, dietary LNT supplementation enhanced ADG, and decreased feed conversion and SUN (*P* < 0.05, Table 1).

**Table 1:** The effect of dietary LNT supplementation and/or RV challenge on growth performance and serum urea nitrogen level in weaned pigs

	− RV		+ RV		*P*-value		
	CON	LNT	CON	LNT	RV	LNT	LNT × RV
1–14 d							
ADFI, g	364.96 ± 29.35	387.99 ± 26.00				0.56	
ADG, g	237.80 ± 20.06	281.25 ± 17.53				0.11	
Feed conversion	1.55 ± 0.04^a^	1.38 ± 0.04^b^				< 0.05	
15–19 d							
ADFI, g	450.11 ± 45.71	466.63 ± 20.89	425.50 ± 42.22	498.55 ± 21.31	0.60	0.13	0.26
ADG, g	304.29 ± 23.92^b^	381.25 ± 25.16^a^	239.58 ± 28.40^c^	354.58 ± 18.88^b^	0.08	< 0.05	0.45
Feed conversion	1.48 ± 0.11^b^	1.24 ± 0.07^c^	1.82 ± 0.10^a^	1.50 ± 0.05^b^	< 0.05	< 0.05	0.67
SUN, mmol/L	6.09 ± 0.05^c^	5.65 ± 0.07^d^	7.35 ± 0.18^a^	6.51 ± 0.09^b^	< 0.05	< 0.05	0.36

*− RV* infusing the essential medium, *+ RV* infusing the porcine rotavirus, *CON* basal diet, *LNT* LNT-supplemented diet, *ADFI* average daily feed intake, *ADG* average daily gain, *SUN* serum urea nitrogen

^a, b, c^ Mean values within a row with different superscript letters were significantly different (*P* < 0.05)

**Fig. 1:**
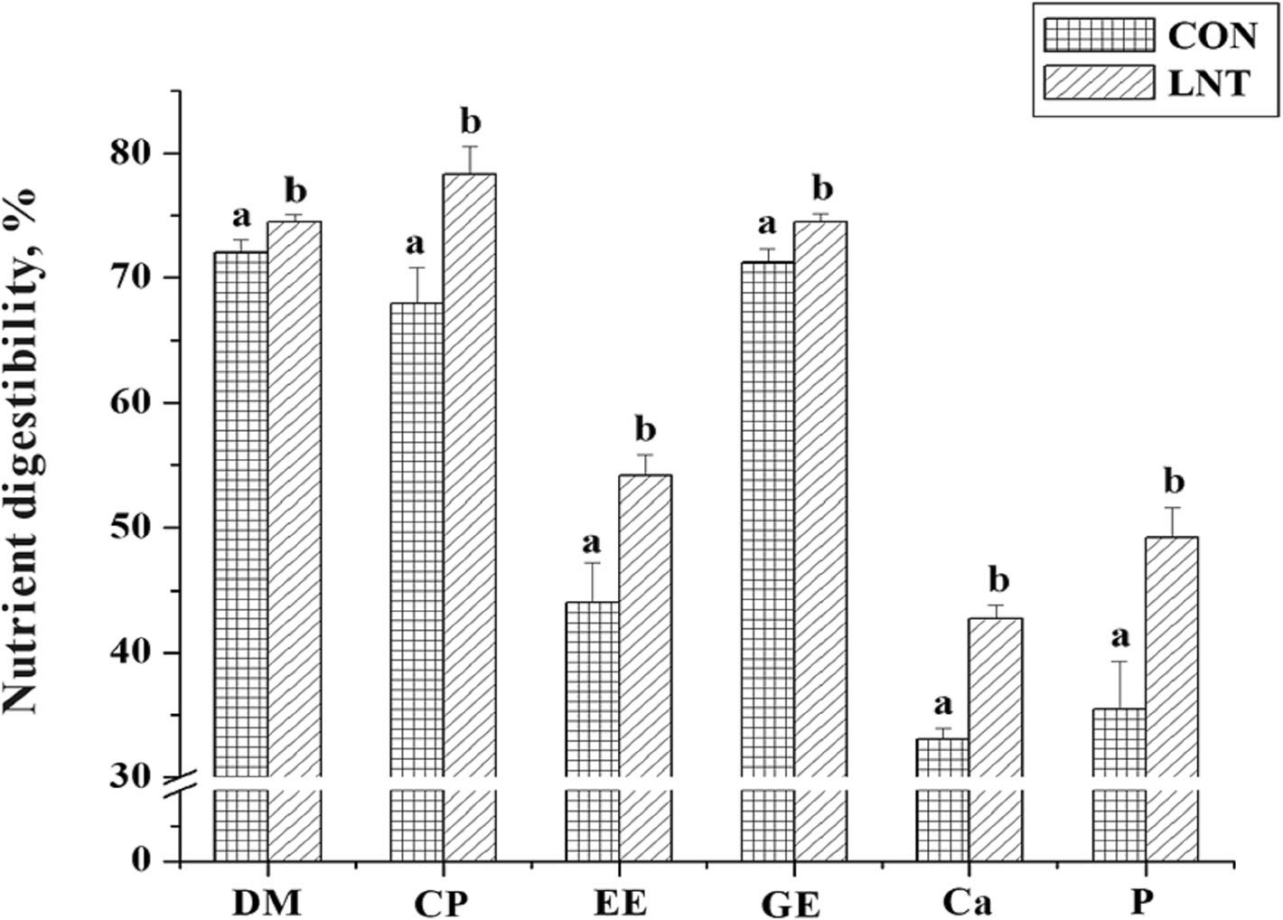
The effect of dietary LNT supplementation on nutrient digestibility of weaned pigs. CON, basal diet; LNT, LNT-supplemented diet; DM, dry matter; CP, crude protein; EE, ether extract; GE, gross energy; Ca, calcium; P, phosphorus. Values were means ± SE (*n* = 14). Values with different superscript letters in the same index were significantly different (*P* < 0.05)

Before RV challenge, dietary LNT supplementation increased the digestibility of DM, CP, EE, GE, Ca, and P in weaned pigs (*P* < 0.05, Fig.1).

### The diarrhea status and RV-ab and/or NSP4 levels of serum and jejunal mucosa in weaned pigs

**Fig. 2:**
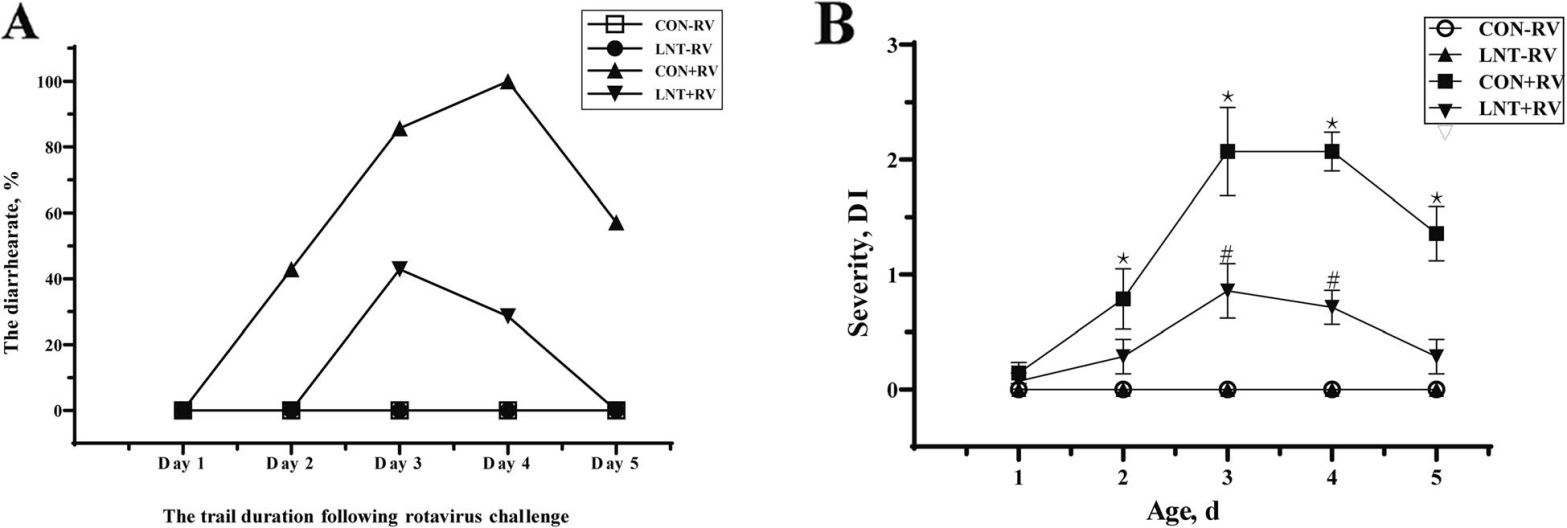
The diarrhea rate (**a**) and severity (**b**) of weaned pigs after rotavirus challenge. − RV, infusing the essential medium; + RV, infusing the porcine rotavirus; CON, basal diet; LNT, LNT-supplemented diet. In (**b**), values were means ± SE (*n* = 7), and values with different symbols in the same day were significantly different (*P* < 0.05)

Infusing the essential medium did not induce diarrhea, but oral RV gavage led to diarrhea in weaned pigs (Fig.2). RV-Ab and/or NSP4 levels of serum and jejunal mucosa in weaned pigs were increased by RV challenge (*P* < 0.05, Table 2). Additionally, in the weaned pigs infused with RV, LNT administration decreased diarrhea rate (Fig.2a), inhibited diarrhea severity (*P* < 0.05, Fig.2b), and reduced NSP4 levels of jejunal mucosa (*P* < 0.05, Table 2), further more enhanced serum RV-Ab level (*P* < 0.05, Table 2). In Fig.2a, it could be also found that time of diarrhea onset in the RV-infected pigs with LNT diet is later than that in the RV-infected pigs with basal diet, and dietary LNT supplementation shortened diarrhea duration.

**Table 2:** The effect of dietary LNT supplementation and/or RV challenge on the immunoglobulin, rotavirus-antibody, rotavirus non-structural protein 4 and/or interferon-β concentrations in serum and jejunal mucosa of weaned pigs

	− RV		+ RV		*P* value		
	CON	LNT	CON	LNT	RV	LNT	LNT × RV
Serum							
IgM, mg/mL	0.17 ± 0.02^c^	0.24 ± 0.01^b^	0.26 ± 0.01^b^	0.35 ± 0.03^a^	< 0.05	< 0.05	0.77
IgG, mg/mL	1.26 ± 0.04^b^	1.49 ± 0.10^a^	1.59 ± 0.06^a^	1.64 ± 0.08^a^	0.07	< 0.05	0.25
IgA, μg/mL	68.58 ± 2.50^c^	73.23 ± 1.54^b^	76.25 ± 1.21^ab^	81.24 ± 1.21^a^	< 0.05	< 0.05	0.92
RV-Ab, ng/L	17.23 ± 2.07^c^	18.29 ± 0.99^c^	22.14 ± 1.07^b^	26.79 ± 1.15^a^	< 0.05	< 0.05	< 0.05
Jejunal mucosa							
NSP4, ng/mg protein	8.60 ± 0.61^c^	8.01 ± 0.56^c^	23.90 ± 0.63^a^	14.15 ± 0.59^b^	< 0.05	< 0.05	< 0.05
sIgA, μg/mg protein	19.25 ± 1.23^b^	25.17 ± 1.52^a^	15.23 ± 0.82^c^	19.72 ± 0.92^b^	< 0.05	< 0.05	0.58
RV-Ab, ng/mg protein	7.71 ± 0.57^b^	8.79 ± 0.72^ab^	8.82 ± 1.04^ab^	9.80 ± 0.55^a^	< 0.05	< 0.05	0.86
IFN-β, pg/mg protein	225.31 ± 26.48^c^	353.65 ± 18.58^b^	401.87 ± 25.57^b^	508.88 ± 16.95^a^	< 0.05	< 0.05	0.64

*− RV* infusing the essential medium, *+ RV* infusing the porcine rotavirus, *CON* basal diet, *LNT* LNT-supplemented diet, *IgA* immunoglobulin A, *IgG* immunoglobulin G, *IgM* immunoglobulin M, *RV-Ab* rotavirus-antibody, *NSP4* rotavirus non-structural protein 4, *sIgA* secretory immunoglobulin A, *IFN-β* interferon-β

^a, b, c^ Mean values within a row with different superscript letters were significantly different (*P* < 0.05)

### Serum immunoglobulin concentrations, and sIgA and IFN-β levels and some gene expressions of jejunal mucosa in weaned pigs

Oral RV gavage increased serum IgM (*P* < 0.05), IgG (*P* = 0.07) and IgA (*P* < 0.05) levels, enhanced IFN-β level of jejunal mucosa (*P* < 0.05), reduced sIgA level of jejunal mucosa (*P* < 0.05), upregulated *TLR3*, *RIG-I*, *MDA5*, *MAVS*, *IFN-β* and *ISG-15* mRNA expressions of jejunal mucosa (*P* < 0.05), and downregulated *pBD1*, *pBD2* and *pBD3* mRNA expressions of jejunal mucosa (*P* < 0.05) in weaned pigs (Tables 2 and 3). Dietary LNT supplementation enhanced serum IgM, IgG and IgA levels (*P* < 0.05), increased sIgA and IFN-β concentrations of jejunal mucosa (*P* < 0.05), and stimulated *TLR3, RIG-I, MDA5, MAVS, IFN-β, ISG-15, pBD1*, *pBD2* and *pBD3* mRNA expressions of jejunal mucosa (*P* < 0.05) in weaned pigs (Tables 2 and 3). Additionally, in the RV-challenged pigs, LNT administration further increased serum IgM concentration, and IFN-β level and mRNA expression of jejunal mucosa, and alleviated the effect of RV challenge on sIgA level, and *pBD1*, *pBD2* and *pBD3* mRNA expressions of the jejunal mucosa (*P* < 0.05, Tables 2 and 3).

**Table 3:** The effect of dietary LNT supplementation and/or RV challenge on mRNA expressions of host defense peptides and immune-related genes in the jejunal mucosa of weaned pigs

	− RV		+ RV		*P*-value		
	CON	LNT	CON	LNT	RV	LNT	LNT × RV
*pBD1*	1.00 ± 0.09^b^	1.22 ± 0.03^a^	0.45 ± 0.04^d^	0.79 ± 0.05^c^	< 0.05	< 0.05	0.30
*pBD2*	1.00 ± 0.06^a^	1.13 ± 0.07^a^	0.51 ± 0.05^c^	0.79 ± 0.03^b^	< 0.05	< 0.05	0.20
*pBD3*	1.00 ± 0.09^b^	1.27 ± 0.06^a^	0.58 ± 0.02^c^	0.94 ± 0.05^b^	< 0.05	< 0.05	0.52
*pBD3*	1.00 ± 0.09^b^	1.27 ± 0.06^a^	0.58 ± 0.02^c^	0.94 ± 0.05^b^	< 0.05	< 0.05	0.52
*TLR3*	1.00 ± 0.12^b^	1.75 ± 0.10^a^	1.69 ± 0.25^a^	1.89 ± 0.21^a^	< 0.05	< 0.05	0.18
*RIG-1*	1.00 ± 0.09^c^	1.14 ± 0.09^bc^	1.34 ± 0.09^ab^	1.94 ± 0.23^a^	< 0.05	< 0.05	0.12
*MDA5*	1.00 ± 0.09^b^	1.49 ± 0.17^ab^	1.61 ± 0.16^a^	1.97 ± 0.25^a^	< 0.05	< 0.05	0.72
*MAVS*	1.00 ± 0.05^b^	2.21 ± 0.16^a^	2.07 ± 0.19^a^	2.32 ± 0.21^a^	< 0.05	< 0.05	< 0.05
*IFN-β*	1.00 ± 0.06^c^	1.42 ± 0.15^b^	1.57 ± 0.08^b^	2.13 ± 0.18^a^	< 0.05	< 0.05	0.59
*ISG-15*	1.00 ± 0.10^c^	1.34 ± 0.15^bc^	1.85 ± 0.25 ^ab^	2.41 ± 0.26^a^	< 0.05	< 0.05	0.58

*− RV* infusing the essential medium, *+ RV* infusing the porcine rotavirus, *CON* basal diet, *LNT* LNT-supplemented diet, *pBD1* porcine beta-defensin 1, *pBD2* porcine beta-defensin 2, *pBD3* porcine beta-defensin 3, *TLR3* Toll-like receptor 3, *RIG-I* retinoic acid inducible protein 1, *MDA5* melanoma differentiation-associated protein 5, *MAVS* mitochondrial antiviral signaling protein, *IFN-β* interferon-beta, *ISG-15* interferon stimulated gene 15

^a, b, c, d^ Mean values within a row with different superscript letters were significantly different (*P* < 0.05)

### Primary characteristics of LNT polysaccharides

Following purification, the purity of polysaccharide from LNT product is 95%. Via analysis, in the HPGPC chromatogram of LNT polysaccharide, there was only one peak, and the Mw was 5303 Da, which indicated that the LNT polysaccharide was possibly pure.

The monosaccharide composition supplies important information for the polysaccharide structure [22], and was also analyzed. Three major monosaccharides including glucose, arabinose and galactose were identified in the LNT polysaccharide while rhamnose, xylose and mannose were also detected. The contents of glucose, arabinose, galactose, rhamnose, xylose and mannose in this polysaccharide were 72.09%, 19.07%, 6.93%, 0.86%, 0.52% and 0.52%, respectively.

### Cell viability

IPEC-J2 cells were exposed to various concentrations (0, 1.5, 3.0, 6.0, 12.0 and 24.0 mg/L) of LNT polysaccharide for 6, 12 and 24 h. During 6 h, the IPEC-J2 cell viability was increased by 1.5 (*P* = 0.07), 3.0 (*P* <  0.05), 6.0 (*P* < 0.01) and 12.0 (*P* = 0.06) mg/L LNT polysaccharide (Table 4). During 12 h, the IPEC-J2 cell viability was only enhanced by 24.0 mg/L LNT polysaccharide (*P* < 0.05, Table 4). After 24 h incubation, 3.0–24.0 mg/L LNT polysaccharide, to some extent, increased IPEC-J2 cell viability, and 24.0 mg/L LNT polysaccharide improved 25.8% viability in IPEC-J2 cells (*P* < 0.01, Table 4). Thus, in the following experiments, the dose and duration of LNT polysaccharide exposure are 24.0 mg/L and 24 h, respectively.

**Table 4:** The effect of LNT polysaccharide on IPEC-J2 cell viability (%)

LNT polysaccharide, mg/L	Treatment duration, h		
	6	12	24
0.0	100.00 ± 2.21	100.00 ± 3.67	100.00 ± 2.35
1.5	108.20 ± 3.44^#^	92.25 ± 2.10	108.06 ± 3.17^#^
3.0	109.72 ± 3.57^*^	102.99 ± 3.76	111.32 ± 3.31^*^
6.0	113.85 ± 1.47^**^	94.53 ± 5.23	117.80 ± 1.24^**^
12.0	109.58 ± 3.36^#^	102.99 ± 2.98	123.36 ± 1.44^**^
24.0	100.86 ± 2.15	115.51 ± 1.94^*^	125.80 ± 1.22^**^

^**#**^*P* < 0.10, ^*^P < 0.05 and ^**^P < 0.01, vs. 0 mg/L LNT polysaccharide group (*n* = 9)

**Fig. 3:**
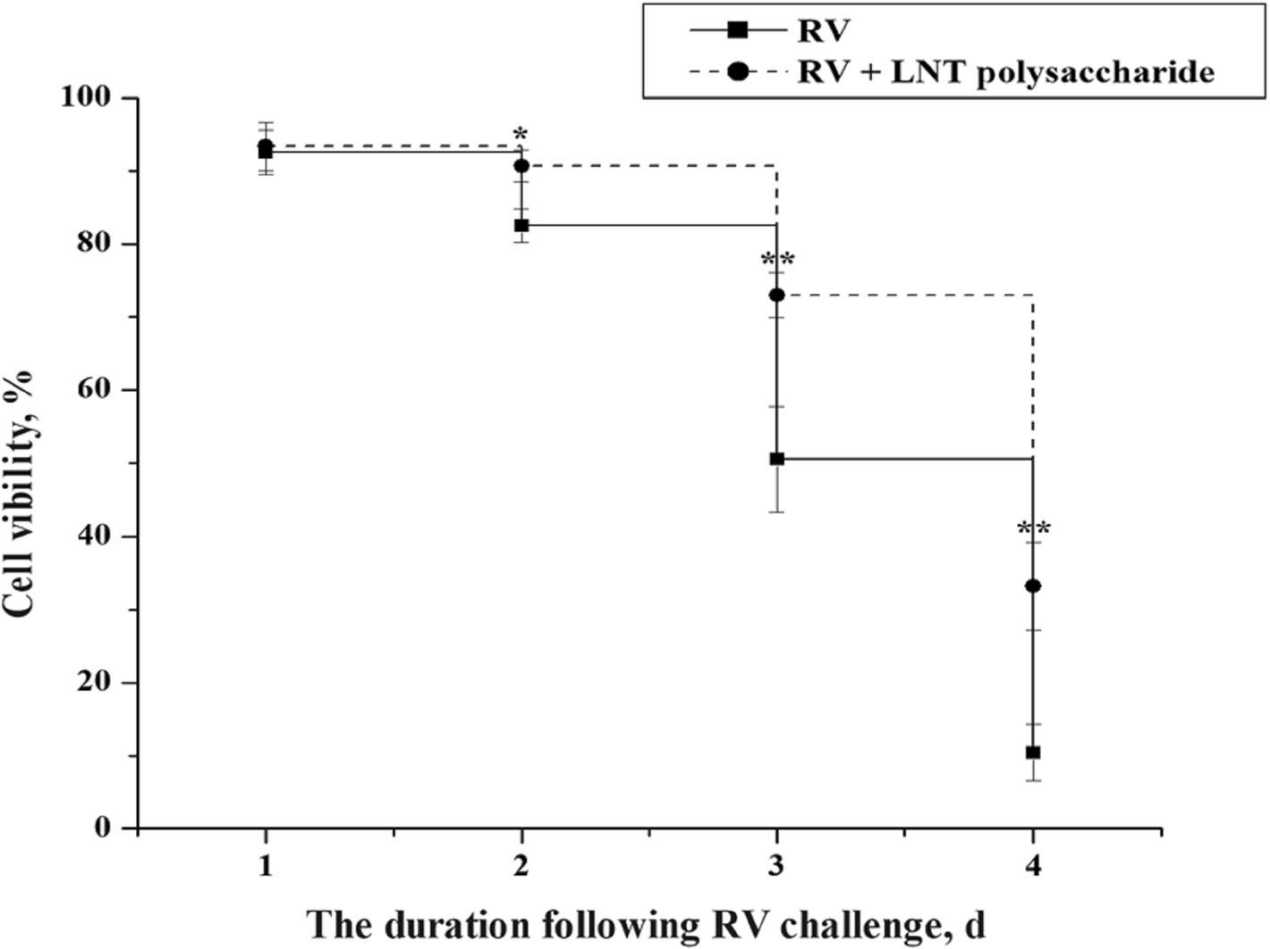
The effect of RV infection on the viability of IPEC-J2 cells with or without LNT polysaccharide supplementation. The cell viability in each time point was calculated on the basis of non-challenge cells with or without LNT polysaccharide exposure. RV, RV-infected cells without LNT polysaccharide exposure; RV + LNT polysaccharide, RV-infected cells with LNT polysaccharide exposure. ^*^P < 0.05 and ^**^P < 0.01, vs. RV-infected cells without LNT polysaccharide exposure in the same time point (*n* = 12)

As shown in Fig.3, the viability of IPEC-J2 cells with or without LNT polysaccharide was decreased by RV infection, which was dependent with exposure duration. However, after IPEC-J2 cells were exposed to 24.0 mg/L of LNT polysaccharide for 24 h, the reduction of IPEC-J2 cell viability induced by RV challenge was alleviated in 2–4 d (*P* < 0.01 or *P* < 0.05, Fig.3).

### The NSP4 concentration in cell supernatants

**Fig. 4:**
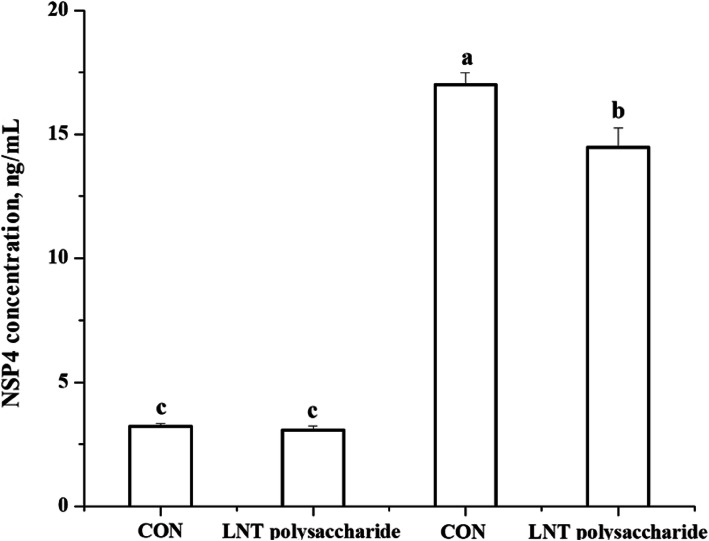
The RV NSP4 levels in IPEC-J2 cell supernatants with or without LNT polysaccharide supplementation following RV challenge. CON, no LNT polysaccharide; LNT polysaccharide, LNT polysaccharide exposure; CON + RV, rotavirus challenge without LNT polysaccharide exposure; LNT polysaccharide + RV, rotavirus challenge with LNT polysaccharide exposure; NSP4, non-structural protein 4. Values with different letters are significantly different (*P* < 0.05, *n* = 6)

With RV challenge, the RV NSP4 level in IPEC-J2 cell media was increased (*P* <  0.05, Fig.4). However, the addition of LNT polysaccharide reduced the RV NSP4 concentration in IPEC-J2 cell media after RV infection (*P* < 0.05, Fig.4).

### The mRNA expressions of immune-related genes in IPEC-J2 cells

**Fig. 5:**
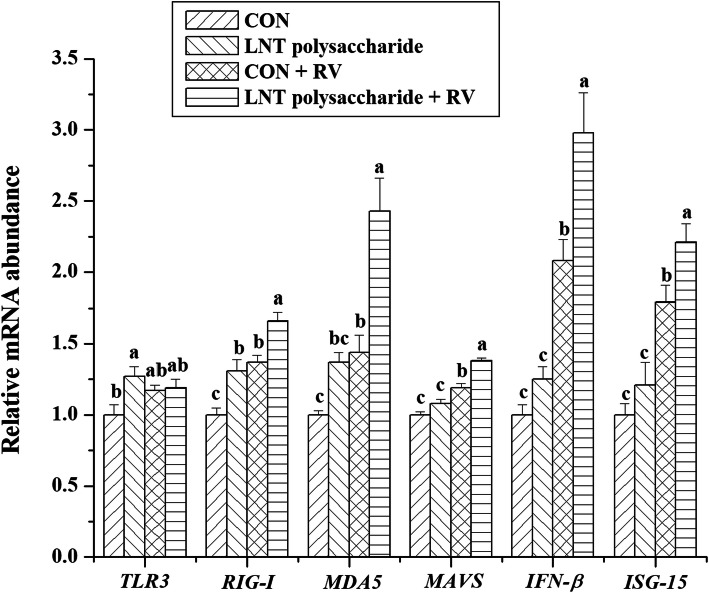
The effects of LNT polysaccharide and RV infection on the mRNA expressions of some pattern recognition receptors in IPEC-J2 cells. CON, no LNT polysaccharide; LNT polysaccharide, LNT polysaccharide exposure; CON + RV, rotavirus challenge without LNT polysaccharide exposure; LNT polysaccharide + RV, rotavirus challenge with LNT polysaccharide exposure; TLR3, Toll-like receptor 3; RIG-I, retinoic acid inducible protein 1; MDA5, melanoma differentiation-associated protein 5; MAVS, mitochondrial antiviral signaling protein; IFN-β, interferon-beta; ISG-15, interferon stimulated gene 15. Values with different letters are significantly different (*P* < 0.05, *n =* 6)

**Fig. 6:**
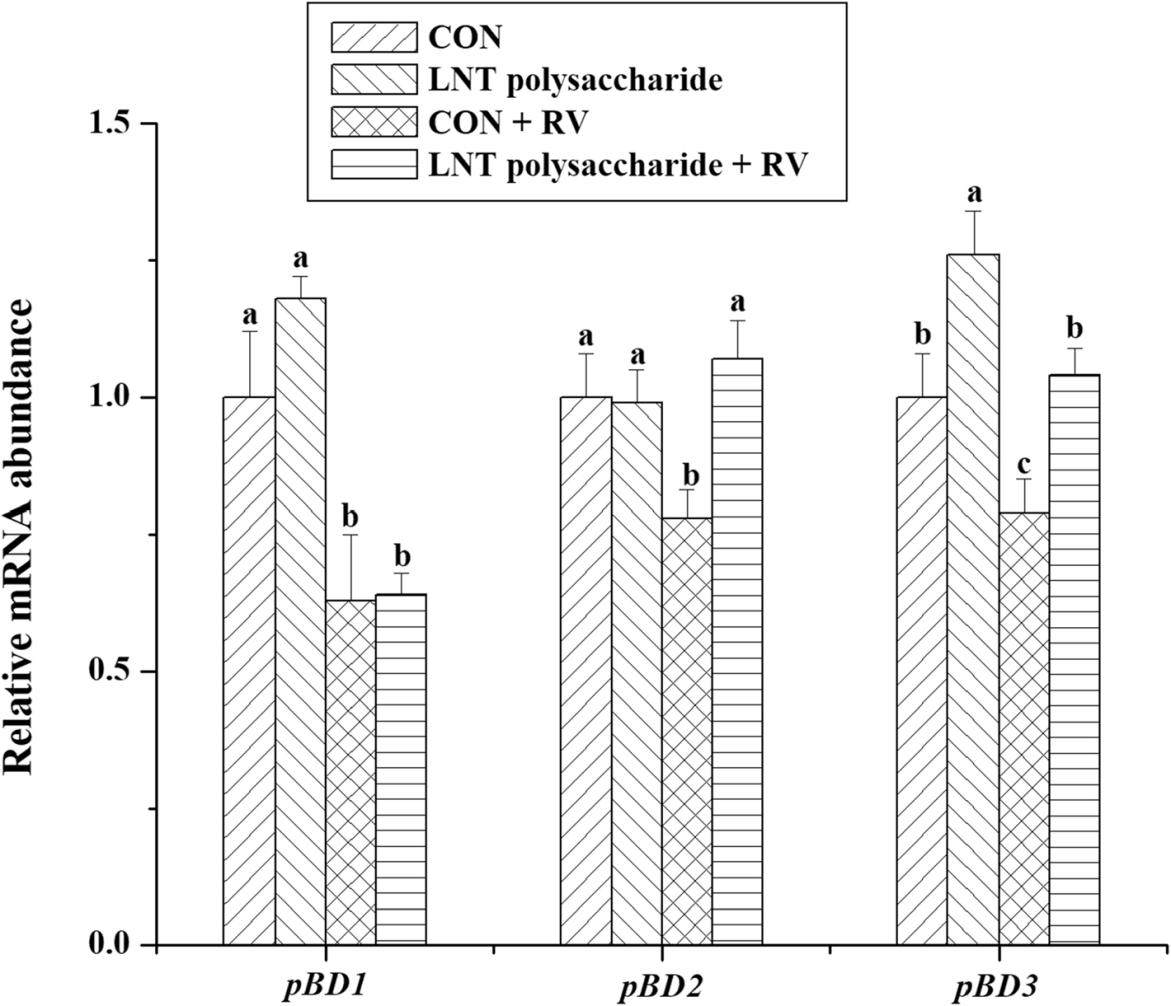
The effects of LNT polysaccharide and RV infection on the mRNA expressions of porcine β-defensins in IPEC-J2 cells. CON, no LNT polysaccharide; LNT polysaccharide, LNT polysaccharide exposure; CON + RV, rotavirus challenge without LNT polysaccharide exposure; LNT polysaccharide + RV, rotavirus challenge with LNT polysaccharide exposure; pBD1, porcine beta-defensin 1; pBD2, porcine beta-defensin 2; pBD3, porcine beta-defensin 3. Values with different letters are significantly different (*P* < 0.05, *n =* 6)

The effects of LNT polysaccharide and RV infection on the mRNA expressions of immune-relative genes in IPEC-J2 cells were exhibited in Figs.5 and 6. RV challenge stimulated *RIG-I*, *MDA5*, *MAVS*, *IFN-β* and *ISG-15* mRNA expressions (*P* < 0.05), and inhibited *pBD1*, *pBD2* and *pBD3* mRNA expressions (*P* < 0.05) in IPEC-J2 cells. However, LNT polysaccharide could improve *pBD2* and *pBD3* mRNA expressions (*P* <  0.05), and further up-regulate *RIG-I*, *MDA5*, *MAVS*, *IFN-β* and *ISG-15* mRNA expressions (*P* <  0.05) in the RV-infected IPEC-J2 cells.

## Discussion

Diarrhea of piglets has been a major problem in the pig industry, which can lead to high mortality of piglets in china. RV is one of the main viral pathogens that cause diarrhea and death in piglets. Many experiments have found that RV can decrease growth performance, induce diarrhea, and impair immune function in children and young animals [25–27], which was consistent with the results of this study. In addition, our study also showed that RV infection increased RV-Ab and/or NSP4 levels in serum and jejunal mucosa of weaned pigs, and induced the decreasing cell viability and the increasing generation of NSP4 in IPEC-J2 cells. These results demonstrated that the *in vivo* and *in vitro* models of RV infection in weaned piglets and IPEC-J2 cells were successfully implemented, respectively.

In this study, dietary LNT supplementation could reduce feed conversion of weaned pigs, which is consistent with previous studies [28]. And we also found that LNT relieved the effect of RV infection on ADG and feed conversion of weaned pigs. The digestion, absorption and utilization of nutrients are important factors that affect growth performance, which, to some extent, can be evaluated by nutrient digestibility and blood urea nitrogen level. The previous study has shown that dietary LNT supplementation could increase the nutrient digestibility of pigs [29], which is consistent with the results of our study. Moreover, in the present study, supplementing LNT in diets might also alleviate the increasing SUN level in piglets with or without RV infection. Thus, it is possible that LNT promoting the growth of piglets should be relative with the increasing digestion, absorption and utilization of nutrients.

The integrity of intestinal epithelial cells is very important to the digestion and absorption function. It is well-known that surface area of intestinal villi is associated with nutrient digestion and absorption. Our previous study has shown that dietary LNT supplementation improved the morphology of jejunal mucosa (especially the increasing villous height) in the normal and RV-infected weaned pigs [12]. Moreover, in present study, we also found that LNT polysaccharide enhanced the viability of IPEC-J2 cells, and alleviated the negative effect of RV challenge on the viability of IPEC-J2 cells. Except for gut morphology, Xue et al. showed that dietary LNT supplementation increased the activities of some digestive- and absorptive-related enzymes in jejunum of pigs [29]. These, to some extent, illustrated the reason that LNT improved nutrient digestibility.

As one of the non-structural proteins in RV, NSP4 is an important factor that RV induces diarrhea in human and animals. It has been considered as a good marker of RV infection [30]. In this study, RV infection enhanced NSP4 levels, but LNT or LNT polysaccharide administration decreased NSP4 levels in jejunal mucosa of weaned pigs and IPEC-J2 cells. These demonstrated that LNT (including LNT polysaccharide) had the potential of inhibiting RV invasion in gut.

Concentrations of LNT polysaccharide ranged from 1.5 to 24.0 mg/L were chosen in our study, which are in accordance with the levels probably encountered in the gastrointestinal tract of piglets after feed intake in animal breeding experiments. Based on the water content in the digesta of small intestine and the recommending dose of LNT product (purity of polysaccharide is 30%) in feeds, the concentrations of LNT product should be 4.5–72.0 mg/L. Finally, the effective dose of LNT polysaccharide (purity of polysaccharide is 95%) in cell experiments was about 1.5–24.0 mg/L.

Immune function can withstand the pathogen, which is very pivotal to maintain gut health of animals and human. Many researches have reported that LNT can regulate immune function [31–33]. Immunoglobulin levels are the important index of humoral immunity [34]. In many *in vivo* and *in vitro* studies, LNT can regulate the immunoglobulin levels [35–38]. This study reported that dietary LNT supplementation could increase serum IgA, IgM and IgG levels in weaned piglets, and further increase IgM level of serum and RV-Ab level of serum and jejunal mucosa in RV-infected piglets. Besides these, we also found that LNT administration restored the effect of jejunal mucosal sIgA level, which is considered as the important content of intestinal immunologic barrier [39, 40]. These demonstrate that LNT improved the growth and health, at least partially, via increasing the humoral immunity of body.

As a kind of type I interferon, IFN-β can stimulate the productions of antiviral protein in cells via up-regulating expressions of some genes (such as *ISG-15*), which reduces virus replication. Then, it was considered as antiviral agents [41]. Under some disease condition (i.e. human immunodeficiency virus, HIV), LNT induces interferon secretion [42, 43]. The current study also showed that, under RV infection condition, LNT administration further increased IFN-β level and mRNA expression in the jejunal mucosa of weaned pigs, and *IFN-β* and *ISG-15* mRNA expressions in IPEC-J2 cells and jejunal mucosa of weaned pigs. Thus, dietary LNT supplementation alleviating RV-induced diarrhea could be associated with IFN-β-increasing antivirus capacity.

When RV invades gut and cells via pathogen-associated molecular pattern (PAMPs), these PAMPs are sensed by pattern recognition receptors (PRRs) of host [44]. These PRRs, such as Toll-like receptors (TLRs), RIG-I-like receptors, are expressed on many cells (including immune cells, epithelial cells) [45, 46]. Following PAMPs’ stimulation, host immunity (PRRs and their related signaling pathways) will be activated, and lead to the transcription and synthesis of many cytokines (especially, type I interferon). This will efficiently inhibit pathogen invasion [47]. In our study, RV infection, to some extent, up-regulated *TLR3*, *RIG-I*, *MDA5* and *MAVS* mRNA expressions in IPEC-J2 cells and jejunal mucosa of weaned pigs, which might induce host anti-viral immunity. Additionally, we found that although LNT administration upregulated the mRNA expressions of *TLR3, RIG-I, MDA5* and *MAVS* of jejunal mucosa in weaned pigs, LNT polysaccharide only further stimulated *RIG-I*, *MDA5* and *MAVS* mRNA expressions, and did not affect *TLR3* mRNA expression in RV-infected IPEC-J2 cells. These also showed that LNT polysaccharide increased anti-viral function of type I interferon via regulating RIG-I/MDA5/MAVS pathways, which could be not associated with TLR3 and its relative pathways.

Host defense peptides (HDPs) also are an important part of innate immunity, which are the barriers for preventing invasion of pathogens [48]. This process is derived from HDPs regulating inflammatory cytokines and responses via some signaling pathways, such as Toll-like receptors 4, mitogen-activated protein kinase and nuclear factor-kappa B signaling pathways [49–52]. And the *HBD-2* mRNA expression in pulmonary epithelial cells could be induced by lentinan in a concentration- and time-dependent manner [53]. The current study showed that RV infection decreased the mRNA expressions of *pBD1*, *pBD2* and *pBD3* in jejunal mucosa of piglets and IPEC-J2 cells, but LNT administration relieved the effect of RV infection on *pBD1*, *pBD2* and *pBD3* mRNA expressions in jejunal mucosa of piglets, and *pBD2* and *pBD3* mRNA expressions in IPEC-J2 cells. About the different results between *in vivo* and *in vitro* trials, it was possibly derived from LNT contents and HDPs’ generation mechanisms. Thus, LNT improving immune function of RV-infected host could be due to the stimulation of HDPs in intestinal mucosa.

## Conclusions

In summary, under normal or RV-challenge conditions, dietary LNT supplementation improved growth performance and decreased diarrhea, which could be due to the increase of nutrient digestibility and immunity in weaned pigs. Via purity of LNT polysaccharide and *in vitro* trials, LNT administration enhanced gut anti-viral immunity possibly via stimulating pattern recognition receptor pathway (such as RIG-I/MDA5/MAVS pathways) and HDPs’ generation. In addition, based on the similarity of physiology between pigs and humans, LNT may be used to prevent and cure rotavirus infection in young children.

## Supplementary Information

**Additional file 1. Table 1** The composition and nutrient levels of basal diets. **Table 2** Primer sequences used for real-time PCR.

## Data Availability

The datasets used and/or analyzed during the current study are available from the corresponding author on reasonable request.

## Abbreviations

LNT: Lentinan
RV: Rotavirus
TCID_50_: Tissue culture infective dose 50
ADG: Average daily gain
ADFI: Average daily feed intake
SUN: Serum urea nitrogen
RV-Ab: Rotavirus antibody
Ig: Immunoglobulin
IFN-β: Interferon-beta
Mw: Molecular weight
HPGPC: High performance gel permeation chromatography
NSP4: Non-structural protein 4
TLR3: Toll-like receptor 3
RIG-I: Retinoic acid inducible protein 1
MDA5: Melanoma differentiation-associated protein 5
ISG-15: Interferon stimulated gene 15
MAVS: Mitochondrial antiviral signaling protein
