# Supplementary material for: Lentinan administration alleviates diarrhea of rotavirus-infected weaned pigs via regulating intestinal immunity
Source: J Anim Sci Biotechnol. 2021 Mar 9;12:43. doi: 10.1186/s40104-021-00562-6 (PMC7945689; doi:10.1186/s40104-021-00562-6)
Supplement: Supplementary file 1 — Additional file 1. Table 1 The composition and nutrient levels of basal diets. Table 2 Primer sequences used for real-time PCR. [file 40104_2021_562_MOESM1_ESM.docx]

Supplementary Table 1 The composition and nutrient levels of basal diets

|  | | Content |
| --- | --- | --- |
| Diet composition, % | |  |
| Corn | 30.18 |  |
| Extruded corn | 29.00 |  |
| Soybean meal | 10.76 |  |
| Extruded soybean | 10.00 |  |
| Wheat bran | 2.00 |  |
| Soybean protein concentrate | 5.00 |  |
| Corn starch | 0.50 |  |
| Whey powder | 4.00 |  |
| Fish meal | 4.00 |  |
| Soybean oil | 1.78 |  |
| CaHPO_4_ | 0.55 |  |
| CaCO_3_ | 0.70 |  |
| NaCl | 0.30 |  |
| L-Lysine·HCl | 0.33 |  |
| DL-Methionine | 0.09 |  |
| L-Threonine | 0.15 |  |
| L-Tryptophan | 0.03 |  |
| Choline chloride | 0.10 |  |
| Vitamin premix^a^ | 0.03 |  |
| Mineral premix^b^ | 0.50 |  |
| Nutrient levels, %^c^ | |  |
| Metabolic energy, MJ/kg | 14.83 |  |
| Crude protein | 20.56 |  |
| Calcium | 0.80 |  |
| Phosphorus available | 0.40 |  |
| Total lysine | 1.35 |  |
| Total methionine and cysteine | 0.74 |  |
| Total threonine | 0.79 |  |
| Total tryptophan | 0.22 |  |

^a^ Provided the following per kg of diet: Vitamin A 8000 IU, Vitamin D_3_ 1500 IU, Vitamin E 25 IU, Vitamin K_3_ 2.0 mg, Vitamin B_1_ 2.0 mg, Vitamin B_2_ 5.0 mg, Vitamin B_6_ 4.0 mg, Vitamin B_12_ 0.1 mg, Niacin 25 mg, Pantothenic acid 12 mg, Folic acid 0.75 mg, Biotin 0.2 mg.

^b^ Provided the following per kg of diet: Fe 100 mg, Cu 6 mg, Mn 4 mg, Zn 100 mg, I 0.14 mg, Se 0.35 mg.

^c^ Nutrient levels were calculated values.

Supplementary Table 2 Primer sequences used for real-time PCR

| Gene | Primer | Nucleotide sequences 5’-3’ |
| --- | --- | --- |
| *β-actin* | Forward | TCTGGCACCACACCTTCT |
|  | Reverse | TGATCTGGGTCATCTTCTCAC |
| *pBD1* | Forward | TCCTTGTATTCCTCCTCA |
|  | Reverse | CAAATCCTTCACCGTCTACCA |
| *pBD2* | Forward | TGTCTGCCTCCTCTCTTCC |
|  | Reverse | ACACGCCTTTATTCCTTA |
| *pBD3* | Forward | CCTTCTCTTTGCCTTGCTCTT |
|  | Reverse | CCTTCTCTTTGCCTTGCTCTT |
| *TLR3* | Forward | CTCGCTGATTCTCCTCTTCTC |
|  | Reverse | TCTCCATTCCTGTCCTGTGA |
| *RIG-I* | Forward | AGGATTCGCAGCGTGGAG |
|  | Reverse | GCAGTGACCGTGACAGACC |
| *MDA5* | Forward | AGACTTGGCTGATCTGTGGC |
|  | Reverse | ACCTGGCACGGAGCTCTTAT |
| *MAVS* | Forward | AGAAGCAGGACACAGAAC |
|  | Reverse | GAAGGAGACAGTCGGAGA |
| *IFN-β* | Forward | CGATACCAACAAAGGAGCAG |
|  | Reverse | GGTTTCATTCCAGCCAGT |
| *ISG-15* | Forward | ACCATTTCTGGCTGACTTTC |
|  | Reverse | ACATAGGCTTGAGGTCATACT |

pBD1, porcine beta-defensin 1; pBD2, porcine beta-defensin 2; pBD3, porcine beta-defensin 3; TLR3, Toll-like receptor 3; RIG-I, retinoic acid inducible protein 1; MDA5, melanoma differentiation-associated protein 5; MAVS, mitochondrial antiviral signaling protein; IFN-β, interferon-beta; ISG-15, interferon stimulated gene 15.
